# Components of acquisition-to-acquisition variance in continuous arterial spin labelling (CASL) imaging

**DOI:** 10.1186/1471-2202-11-30

**Published:** 2010-03-02

**Authors:** Roberto Viviani, Petra Beschoner, Hanna Lo, Nadine Osterfeld, Jan Thöne, Eun-Jin Sim

**Affiliations:** 1 Department of Psychiatry and Psychotherapy III, University of Ulm, Leimgrubenweg 12, Ulm, Germany; 2 Department of Psychosomatic Medicine and Psychotherapy, University of Ulm, Hochsträß, Ulm, Germany; 3 Department of Neurology, St Josef Hospital/Ruhr-University Bochum, Gudrunstr. 56, Bochum, Germany

## Abstract

**Background:**

Images of perfusion estimates obtained with the continuous arterial spin labelling technique are characterized by variation between single acquisitions. Little is known about the spatial determinants of this variation during the acquisition process and their impact on voxel-by-voxel estimates of effects.

**Results:**

We show here that the spatial patterns of covariance between voxels arising during the acquisition of these images uncover distinct mechanisms through which this variance arises: through variation in global perfusion levels; through the action of large vessels and other, less well characterized, large anatomical structures; and through the effect of noisy areas such as the edges of the brain.

**Conclusions:**

Knowledge of these covariance patterns is important to experimenters for a correct interpretation of findings, especially for studies where relatively few acquisitions are made.

## Background

Arterial spin labelling (ASL, [1]) is a non-invasive technique for the measurement of cerebral blood flow (CBF), enabling investigators to study brain perfusion with magnetic resonance techniques (see [2,3] for general introductions). Recent advances in arterial spin labelling techniques allow the practical acquisition of CBF estimates with wide spatial coverage of the brain using multi-slice or 3D acquisitions [4]. This has made of ASL a very promising technique for the systematic investigation of the physiology and functional determinants of brain perfusion, and of individual differences in baseline CBF at rest [5-7].

Several studies have described the spectral components of time series in ASL data [8-10]. This study is concerned with the spatial patterns of covariation in the residual images of linear models fitted to quantitative perfusion images obtained with the continuous arterial spin labelling (CASL) technique, as implemented by [11]. A previous study investigated the principal components associated to subject-to-subject spatial variance, and described acquisition-to-acquisition variance [12]. Study of spatial covariation is of interest for two reasons. Firstly, it gives insight on the interplay of brain physiology with respect of vascularisation, and the signal acquired with this imaging technique. Secondly, knowledge of spatial covariation is important to experimenters because it constitutes a violation of the stationarity assumption on the random field that models spatial covariation of residuals after smoothing [13,14]. Under this assumption, residuals are spatially distributed like smoothed white noise. Concretely, the violation of this assumption means that under the null hypothesis the estimated effects are likely to reflect the pattern determined by the most important patterns of covariation, rather than a random set of blobs scattered across the brain. Therefore, when inspecting images of the estimated effects, knowledge of the form these images are likely to take even if the null hypothesis is true can assist in evaluating the spatial patterns produced by an experiment or an observational study, and distinguishing between sources of variation due to vascularisation and those due to the variable of interest.

**Figure 1:**
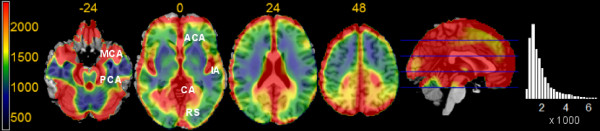
**Acquisition-to-acquisition variance maps**. Acquisition-to-acquisition variance, axial slices, overlaid on a template brain. Very large variance (in red) is present at the edges of the brain and in correspondence of the course of large vessels (MCA: middle cerebral artery; PCA: posterior cerebral artery; ACA: anterior cerebral artery and frontal arteries; IA: insular arteries; CA: middle occipital and choroidal arteries; RS: sinus rectus). Most of the brain parenchyma, in contrast, displays lower variance (in yellow, green, and blue), especially in white matter. On the right, the histogram of the variance values shows a long right tail; isolated values reach *σ*^2 ^= 30 000.

Here, we refer to acquisitions as to images of CBF values computed from applying a compartment model to two scans, one with and one without labelled spins [11]. In a study of baseline perfusion at rest, acquisition-to-acquisition variance arises from variation from one estimated perfusion image to the other within subjects. In a study in which participants carry out a task in the scanner, acquisition-to-acquisition variance is constituted by the residual variance arising from one estimated perfusion image to the other within experimental conditions/factor levels. Figure 1 displays maps of acquisition-to-acquisition variance, collected from participants resting quietly in the scanner with closed eyes for 8 min, and therefore originated from a study of the first type. One can see that this variance shows differences of one order of magnitude across the brain, and that these differences follow anatomical features such as the course of large vessels [12]. High variance also occurs at the edges of the brain and in ventricles, where the model used to estimate perfusion may not hold (because quantified CBF is estimated from a ratio, misspecification of the model may amplify the variation in the denominator). Because the reasons for high variance in these areas and near large vessels may differ, it is conceivable that spatial covariance may show different intensities depending on the mechanism at the basis of variance. Specifically, we would expect high variance at the edges of the brain to arise because of high variation from one voxel to the next leading to low spatial covariance, while near large vessels groups of voxels may tend to assume largely different values from one acquisition to the other, giving rise to larger spatial correlations.

In this study, patterns of spatial covariation will be identified by carrying out a principal component analysis of the estimated acquisition-to-acquisition variance shown in Figure 1. The sample consists of a total of 13 680 images collected from 228 participants, and because of its size should yield a quite reliable eigendecomposition. To characterize the origin of these components further, the relation of the main components of variation with global CBF levels will be investigated.

## Results

### Principal component analysis

**Figure 2:**
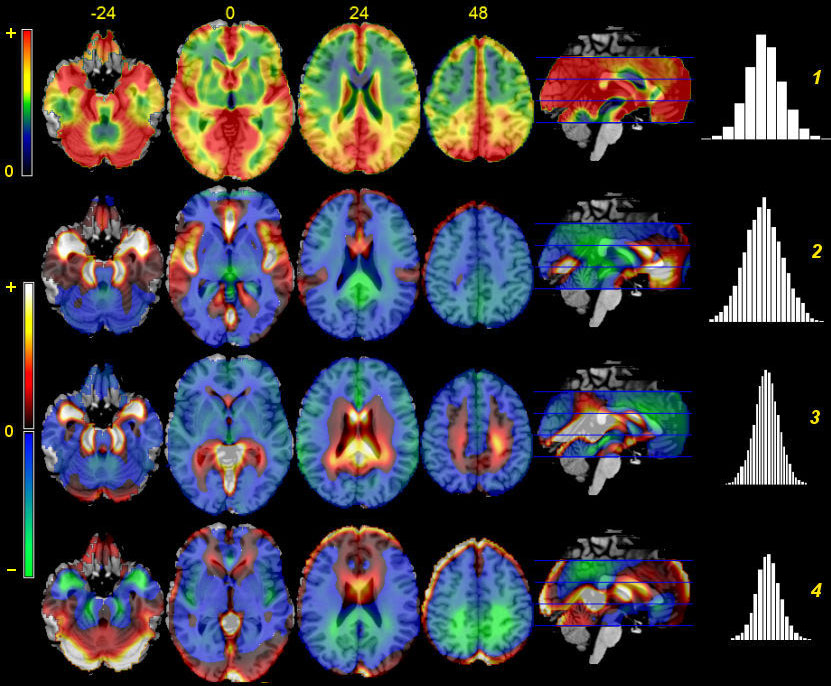
**Principal components maps**. From top to bottom, component images of the first four components of acquisition-to-acquisition variance, overlaid on template brains. On the right, histograms of the component scores (unitless). The scale and sign of these images is arbitrary. The coefficients of the first component are positive across the brain; for better contrast, a different colour scale was used than for the remaining components.

**Figure 3:**
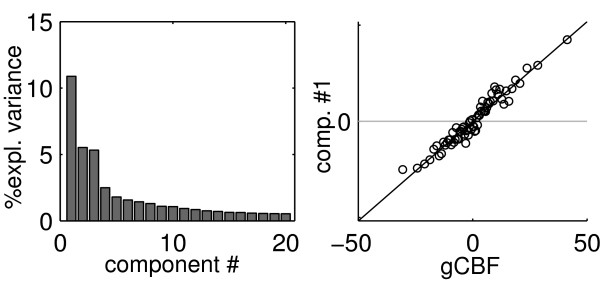
**Screeplot**. On the left, screeplot of the first 20 principal components. On the right, scatterplot of the first component score on global CBF values (differences from the mean subject global CBF in ml/100 gr/min). Component scores refer to individual volumes. To increase the clarity of the plot, global CBF was ranked, and sampled at intervals of 200 rank scores.

Figure 2 shows the first four principal spatial pattern of acquisition-to-acquisition variation obtained through principal component analysis. The relative weights of the first 20 components, expressed by the proportion of variance captured in the data, are displayed in Figure 3, left. Even if individual acquisition images may be characterized by variance at high spatial frequency [12], the common modes of variation displayed in these images tend to be spatially smooth (very similar components, not shown here for brevity, were obtained by analysing non-smoothed data).

The first component explained about 10% of the variance, and contained variation shared by all voxels, shown by the coefficients of the component being of the same sign over the whole volume. This mode of variation was determined by the perfusion level of the acquisition in the whole volume, as is shown by the high correlation of the score of this component with global CBF values (*R*^2 ^= 94%, *p *< 0.001, Figure 3). Areas of high acquisition-to-acquisition variance, visible in Figure 1, were represented in the spatial pattern of this component, which loaded with different intensity depending on overall acquisition-to-acquisition variance. Taken together, the evidence presented in these Figures shows that when global CBF of the acquisition is high, the computed regional CBF at the large vessels is even higher than the average level; furthermore, also perfusion in the cortex increases more than in the white matter. However, the variation of the first component appears to affect the calcarine and medial occipital cortex more than other cortical areas and as anticipated by their vicinity to large vessels (slices at *z *= 0 and 24, top row, Figure 2). Furthermore, the high variance at the edges of the brain and in ventricles of Figure 1 is underrepresented. Outside the brain parenchyma, high spatial covariation is observed in the space between the hemispheres and in the Sylvian scissure.

The second and third components, each accounting for about 5% of the variance, captured variation located along the course of main vessels. The dissociation between anterior and posterior areas visible in the comparison between these two components reflects the main subdivision of branches stemming from the internal carotids and the vertebral artery. The fourth component, accounting for about 2.5% of the overall variance, reflected dissociation between the brain parenchyma as a whole and the high-variance edges. In these components, association with global CBF was negligible (*R*^2 ^was 1.5, 2.4 and 0.3% for the second, third, and fourth components), in contrast to the first component. No component up to the 10^th ^was noted to reflect right-left asymmetries in perfusion.

## Discussion

In this study, the pattern of spatial covariation in the acquisition-to-acquisition variance in CASL images was investigated using principal component analysis in a large sample of images. The results obtained here reflect the effectiveness and accuracy with which this technique could be implemented in our laboratory, and are certainly of limited generalizability to future techniques that may seek a more accurate estimation of CBF. However, the CASL imaging protocol implemented here represents a pragmatic approach to CBF estimation (that is frequently adopted in fMRI ASL studies), and its adoption may be informed by the results presented here.

The first component of variation captured changes in the overall intensity of the quantitative CBF estimate in each acquisition. These changes affected gray more than white matter and large vessel areas more than the rest of the brain parenchyma. An important aspect of this component of variation is its high correlation with global perfusion levels (which does not arise by necessity). Even if the spatial pattern of covariance is somewhat different, this component is in this respect analogous to the first component obtained by the principal component analysis of subject-to-subject variance, which also captures changes of global perfusion levels [12]. The finding that the first component is associated with global perfusion levels in both subject-to-subject and acquisition-to-acquisition sources of variance suggests that many factors, now affecting variation between subjects, now affecting variation between acquisitions, may have a common effect on global perfusion levels. What one then sees is that differences in global perfusion levels affect brain regions with distinct but characteristic spatial patterns, depending on whether these factors have acted between acquisitions or between subjects. This observation is relevant for the application of procedures that, with the aim of reducing this source of variance and increase testing power, correct for global CBF values [15-17].

The second and third components reflected a vascular factor, consistent with the predominant dissociation between the anterior and posterior regions due to the two main sources of arterial supply, internal carotids and cerebral artery. This source of variation may ensue from labeled spins that have not yet reached the capillary bed, or from interactions between the timing of the labeling pulse and the rhythm of blood flow. These components reflect the interplay of brain physiology, specifically of large vessels, and the signal acquired with this imaging technique. In contrast, variation at the edges of the brain is underrepresented in covariance patterns, loading strongly only on the fourth component. This is consistent with the observation of the noisy character of the data at the brain edges.

The relative importance of the first three components, accounting together for over 20% of the total variance, shows that residual variation across voxels in this type of images is affected by important violations of the stationarity assumption on the Gaussian random field modeling residual variation. Furthermore, the spatial pattern of these components follows anatomical boundaries, such as the anterior and medial temporal lobe, the medial occipital and calcarine cortex, the insula (especially posteriorly), and the subgenual portion of the anteromedial prefrontal cortex. When averaging these images, some combination of these patterns, apparently following anatomical structures, may arise just by chance, as was demonstrated in the case studies. The anteromedial part of the temporal lobe and the lower half of the medial aspect of the brain hemispheres appear to be particularly affected. Note also that the apparently homogenous high variance of the medial aspect of the brain hemispheres visible in the sagittal slice of Figure 1 is in fact produced by the superposition of spatially characterized patterns.

## Conclusions

Using principal component analysis, we investigated the patterns of spatial covariation arising from changes in the global estimated perfusion levels and their interplay with vascular anatomy.

## Methods

### Data acquisition

Perfusion images at rest (8 min., 120 scans giving 60 acquisitions of perfusion estimates) were acquired using continuous arterial spin labeling [11] from 228 right-handed participants (101 males) aged between 17 and 52 years at the time of the scan (mean age 24.7, std. dev. 5.4) who gave informed consent. The study protocol was approved by the local ethical committee and was in compliance with national legislation and the Code of Ethical Principles for Medical Research Involving Human Subjects of the World Medical Association. Exclusion criteria were neurological or medical conditions, use of medication, or a history of mental illness, and subclinical structural abnormalities.

All magnetic resonance imaging (MRI) data were obtained with a 3-Tesla Magnetom Allegra (Siemens, Erlangen, Germany) MRI system equipped with a head volume coil. All participants were scanned at the Department of Psychiatry of the University of Ulm. A standard T2-weighted structural brain scan from the clinical screening routine in use in our hospital (TR 4120, TE 82) was taken on all participants to exclude subclinical structural abnormalities. A continuous arterial spin-labelling technique was used as described in ref. [11]. Interleaved images with and without labelling were acquired for 8 min by using a gradient-echo echo-planar imaging (EPI) sequence with a field of view of 22 cm. Image size was 64 × 64 × 15 voxels, slice thickness 6 mm with a gap of 1.5 mm, giving a voxel size of 3.44 × 3.44 × 7.50 mm. The images were acquired with TR 4000, TE 17, anterior-to-posterior phase encoding, a flip angle of 90°, and a bandwidth of 3005 Hz/Pixel. A delay of 1 sec was inserted between the end of a 2 sec labelling pulse and image acquisition to reduce transit artefacts. The SPM2 package was used (Wellcome Department of Cognitive Neurology, London; online at http://www.fil.ion.ucl.ac.uk) for realignment and stereotactic normalization to an EPI template (Montreal Neurological Institute, resampling size: 2 × 2 × 2 mm). Reconstruction of CBF values at each voxel was obtained using the Perf_resconstruct_V02 SPM add-on software by H. Y. Rao and J. J. Wang, from the Department of Radiology and Center for Functional Neuroimaging at University of Pennsylvania (online at http://www.cfn.upenn.edu/perfusion/software.htm). The software implements eq. (1) of ref. [18], or, equivalently, eq. (1) of ref. [11]. The 'simple subtraction' method was used. No scaling procedures such as 'grand mean scaling' were applied to the data. All volumes were smoothed using an isotropic Gaussian kernel of full width half-maximum (FWHM) of 6 mm prior to the principal component analysis. An explicit mask was obtained by combining an *a priori *thresholded tissue probability maps provided by the SPM package at 0.25 for gray or white matter with another mask thresholding the standard deviation of the mean images to less than 25 (as described in ref. [12]). Furthermore, slices lower than *z *= -24 mm. were excluded, since very low slices have very large variance in our data (these slices are close to where the labelling pulse was given). We also excluded slices above *z *= 48 mm. to prevent lack of coverage of the top of the brain in some individuals to influence the outcome of the principal component analysis. After masking, each volume contained 158 856 voxels.

### Statistical analysis

The data of the present sample contain two sources of variance: subject-to-subject, and acquisition-to-acquisition. The former is the variance arising from differences in brain perfusion among individuals, while the latter is constituted by variation due to unaccounted effects and experimental error in the data collected within each individual. Formally, in an experiment in which *j *= 1,... *m *baseline perfusion images are acquired in a single session from *i *= 1,... *n *individuals, the data at each voxel may be modelled by a simple random effects ANOVA:

where *y*_*ij *_is the voxel signal, *A*_*i *_is the average regional CBF in subject *i*, and *ε*_*ij *_is an experimental error term at each acquisition containing the unaccounted effects. *A *and *ε *are assumed to be independent random variables with means *μ *and zero, and variance  (the subject-to-subject variance) and *σ*^2 ^(the acquisition-to-acquisition variance), respectively. Voxel-by-voxel estimates of acquisition-to-acquisition variance were obtained by standard ANOVA estimators; in the present case, the estimator of acquisition-to-acquisition variance is given simply by the variance of images  centred relative to the subject mean image.

Principal component analysis was carried out as described in the Appendix of ref. [19] on the estimated images . The analysis was carried out by directly calling the relevant BLAS and LAPACK routines in the version of these packages provided with MATLAB R2006b (The Mathworks, Natick, MA) installed on a machine equipped with a 64-bit Athlon processor (Advanced Micro Devices, Sunnyvale, CA) running Windows XP (Microsoft, Redmond, WA). Data were single-centered voxel by voxel, thus considering voxels to be 'variables' and the average individual volumes to be 'observations' in the usual principal component terminology [20]. A *nm *× *nm *covariance matrix was then computed from the outer product of the data acquired in each voxel. In the present case, *n *= 228 and *m *= 60, giving a covariance matrix of size 13 680 × 13 680. A singular value decomposition of this matrix gave the principal directions of variation in time, i.e. a set of orthonormal vectors of size 13 680. The spatial components ('eigenimages') were obtained as the coefficients of the voxel-by-voxel regression of all volumes on this set of vectors. The principal component analysis also delivers a principal component score, one for each volume. This score is the inner product between the eigenimages and the CBF values in each volume, and represents the extent to which each volume displays the pattern identified by the components. The correlation of this score with global CBF values provides a summary measure of how much the pattern of variation is associated with changes in global perfusion. All images were generated with the software package MRIcroN, obtained from http://www.sph.sc.edu/comd/rorden/mricron/.

## Authors' contributions

RV conceived of the study and its design, carried out scans, wrote software for the statistical analysis, analyzed data, and drafted the manuscript. PB, HL, NO, JT recruited participants, carried out scans, and contributed to writing the manuscript. EJS coordinated the study, carried out scans, and contributed to writing the manuscript. All authors read and approved the final manuscript.
